# Internalization of Exogenous Myelin by Oligodendroglia Promotes Lineage Progression

**DOI:** 10.1002/glia.70132

**Published:** 2026-01-07

**Authors:** Carla Peiró‐Moreno, Juan Carlos Chara, Katy Marshall‐Phelps, Irune Ugarte‐Arakistain, Stefano Calovi, Rafael Gois De Almeida, María Domercq, Carlos Matute

**Affiliations:** ^1^ Department of Neurosciences University of the Basque Country UPV/EHU Leioa Spain; ^2^ Achucarro Basque Center for Neuroscience Leioa Spain; ^3^ CIBERNED‐Instituto de Salud Carlos III Leioa Spain; ^4^ Institute for Neuroscience and Cardiovascular Research, University of Edinburgh Edinburgh UK; ^5^ MS Society Edinburgh Centre for MS Research, University of Edinburgh Edinburgh UK; ^6^ Biobizkaia Health Research Institute Barakaldo Spain

## Abstract

Oligodendrocytes, traditionally recognized for their role in central nervous system myelination, have emerged during the last decades as key participants maintaining brain homeostasis in response to metabolic demands and stress. In addition, injury to myelin prompts a regenerative response that leads to the formation of new myelin sheaths. However, the signals regulating effective remyelination by oligodendrocytes are still not completely understood. Here, we report that oligodendrocytes can internalize exogenous myelin both in vitro and in vivo, which leads to an increase in oligodendroglial lineage progression. RNA sequencing reveals that myelin debris alters the oligodendrocyte transcriptional profile, leading to the suppression of immune‐related pathways and de novo cholesterol and fatty acid biosynthesis, while promoting lipid droplet formation for the storage and processing internalized myelin particles. In primary cultures, myelin exposure increases oligodendrocyte progenitor (OPC) proliferation and overall oligodendroglia lineage progression, accompanied by greater cellular complexity and a larger myelinated area per cell, without altering the relative OPC‐to‐mature oligodendrocyte ratio. Stereotaxic injection of fluorescent myelin into mouse cortex and zebrafish ventricles shows internalization by microglia and, to a lesser extent, by oligodendroglia. Notably, in the zebrafish model, ventricular injections of myelin also increase the number of ventral oligodendrocytes in the spinal cord, further supporting that myelin can promote lineage progression. These findings challenge the classical view that myelin debris intrinsically inhibits oligodendrocyte proliferation, suggesting instead that oligodendrocytes can use myelin to support self‐renewal and maturation across vertebrate species, acting as a trophic factor in the absence of pathological cues.

## Graphical abstract

- Oligodendroglia internalize extracellular myelin debris in vitro and in vivo.
- Internalized myelin modulates lipid metabolism and lipid droplet formation.
- Myelin‐derived signals promote oligodendroglial proliferation and lineage progression.

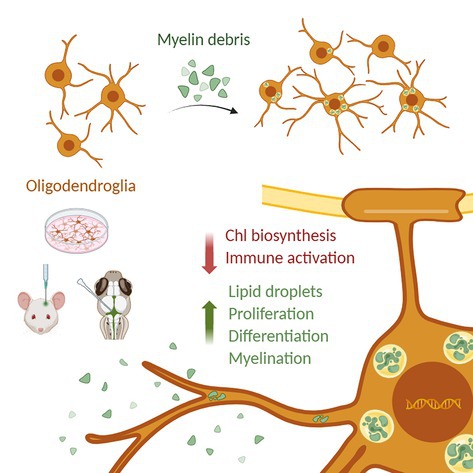

## 1 Introduction

Oligodendrocytes (OLs) are a highly specialized class of glial cells of the central nervous system (CNS), best known for their role in producing myelin sheaths following their differentiation from oligodendrocyte progenitor cells (OPCs) during early development (Bradl and Lassmann 2010; Emery 2010; Nave and Werner 2014). Myelin is a multilamellar, lipid‐rich structure composed of approximately 80% lipids, 20% proteins, and a small fraction of mRNA (Simons and Nave 2016; Stadelmann et al. 2019). Traditionally, OLs were considered primarily responsible for axonal myelination, a process that enables saltatory conduction, in which electrical impulses jump between nodes of Ranvier along the axon, allowing rapid propagation of action potentials (Bradl and Lassmann 2010; Nave and Werner 2014; Simons and Nave 2016). However, research over the last decades has expanded this view, revealing additional mechanisms through which OLs contribute to the maintenance of neuronal function.

Beyond their role in myelination, OLs also play a key role in the metabolic support of axons (Nave et al. 2023; Philips and Rothstein 2017). Neurons have high energy demands due to constant ATP‐dependent activity of ion pumps, synaptic vesicle recycling and generation of membrane potentials (Faria‐Pereira and Morais 2022). Paradoxically, their lack of intrinsic energy reserves makes them reliant on surrounding glial cells for metabolic support. In addition to astrocytes (Tekkök et al. 2005), under homeostatic conditions OLs contribute to this energy supply primarily by transferring lactate to axons through the monocarboxylate transporter 1 (MCT1) (Lee et al. 2012; Rinholm et al. 2011). Furthermore, it has recently been suggested that in response to stress, OLs can modulate their own metabolic machinery to obtain energy from myelin‐derived fatty acids. Genetically starved mice, lacking GLUT1 expression in OLs, exhibit a reduction in the thickness of their myelin sheaths (Asadollahi et al. 2024), a phenomenon that correlates with the loss of myelin in specific brain areas in humans following the extreme physical stress of running a marathon (Ramos‐Cabrer et al. 2025). Likewise, the use of myelin lipids as an energy source has been proposed in the context of aging as a compensatory mechanism for age‐related metabolic decline (Klosinski et al. 2015).

In experimental autoimmune encephalomyelitis, a mouse model of multiple sclerosis (MS), single‐cell RNA analysis demonstrates that OLs exhibit a form of non‐professional phagocytic behavior, upregulating interferon response genes and expressing major histocompatibility complex type 1 and 2 (Falcão et al. 2018). Conventionally, clearance of myelin debris has been attributed to microglia and infiltrating macrophages, which actively engulf and degrade lipid‐rich remnants during injury or disease (Franklin and Ffrench‐Constant 2017; Kent and Miron 2024). In turn, it has been proposed that OL‐lineage cells can internalize extracellular myelin debris, activating memory and effector CD4^+^ cells (Falcão et al. 2018). Therefore, there is emerging evidence that OLs may have alternative roles in pathological conditions.

Further research is needed to delineate the molecular mechanisms involved in this phagocytic response and the extent to which these non‐canonical properties of OLs occur in physiological and pathological conditions. In this study, we demonstrate that oligodendroglia can internalize extracellular myelin debris both in vitro and in vivo and that this process promotes their proliferation and differentiation. By demonstrating the capacity of OLs to engage in debris clearance and that this process is associated with increased proliferation, we highlight the importance of a previously underappreciated aspect of their biology. This insight underscores the evolving view of OLs not just as passive support cells, but as dynamic regulators of CNS integrity and repair.

## 2 Material and Methods

### 2.1 Animals

#### 2.1.1 Rodents

Primary cell culture experiments were conducted using Sprague Dawley rats, sourced from the Animal Facility of the University of the Basque Country (UPV/EHU) (Leioa, Spain). C57BL/6J mice obtained from Jackson Laboratories (Bar Harbor, Maine, USA) were used to perform stereotaxic injections. Animals were housed under standard conditions, maintaining a 12 h light/dark cycle and *ad libitum* access to food and water. Experiments were reviewed and approved by the internal Animal Ethics Committee of the UPV/EHU, according to the guidelines set by the European Communities Council Directive 2010/63/EU.

#### 2.1.2 Zebrafish

Zebrafish ( *Danio rerio* ) were maintained under standard laboratory conditions at the University of Edinburgh, in compliance with UK Home Office regulations (PP0103366) and institutional ethical guidelines. Adult fish were housed on a 14‐h light/10‐h dark cycle. Embryos were raised at 28.5°C in 90 mm Petri dish with 10 mM HEPES‐buffered E3 medium (5 mM NaCl, 0.17 mM KCl, 0.33 mM CaCl_2_, 0.33 mM MgSO_4_), staged by days post‐fertilization (dpf) according to Kimmel et al. (1995), and analyzed up to 4 dpf, prior to the onset of sexual differentiation.

The following transgenic lines were used in this study: *Tg*(*mbp:EGFP‐CAAX*) (Almeida et al. 2011), *Tg*(*mbp:EGFP*) (Almeida et al. 2011), *Tg*(*mpeg1:EGFP*) (Ellett et al. 2011), and *Tg*(*olig1:nls‐mApple*) (Marisca et al. 2020).

### 2.2 Oligodendrocyte Cell Culture

Primary oligodendrocyte cultures were obtained as previously described (Barres et al. 1992), with modifications (Sánchez‐Gómez et al. 2018). Briefly, optic nerves were extracted from P11‐12 rats. After enzymatic and mechanical digestion, cells were filtered and seeded on 1 μg/mL poly‐D‐lysine‐coated (PDL) coatings (Sigma‐Aldrich). Otherwise stated, OLs were maintained in differentiation media, described in Sánchez‐Gómez et al. (2018).

Oligodendrocytes were seeded at 10,000 cells/well for immunocytochemical (ICC), cell viability experiments and on Ibidi μ‐Dishes for time‐lapse imaging. For RNAseq analysis, 300,000 cells/well were seeded. For myelination assays, cells were seeded on coverslips with polycaprolactone nanofibers (Sigma‐Aldrich) at a 20,000 cells/well density and CNTF and NT‐3 were omitted from the medium. Cultures were used after 1 day in vitro (DIV) when almost > 98% cells were O4^+^ oligodendrocytes (Domercq et al. 2005).

### 2.3 Cortical Glial Cell Cultures

Primary mixed glial cultures were obtained from the cortical lobes of P0–2 rats, according to previously described by Mccarthy and De Vellis (1980) with modifications (Sánchez‐Gómez et al. 2018). Briefly, forebrains were dissected to isolate the cortical lobes and after enzymatic and mechanical digestions, cell suspension was seeded in 75 cm^2^ flasks coated with PDL (Sigma‐Aldrich). The resulting glial culture contained OPCs, microglia, and astrocytes. After 14 days, the different cell types were isolated and seeded using different mechanical shakings, based on the different adhesion properties as described in Domercq et al. (2007) for microglial cells and in Sánchez‐Gómez et al. (2018) for OPCs. After detachment and isolation of microglia and OPCs, the astrocyte monolayer was trypsinized with Trypsin–EDTA (0.5 g/L porcine trypsin and 0.2 g/L EDTA, Sigma‐Aldrich) during 7 min and the cell suspension was seeded.

All cell types were seeded on PDL coatings and cell density was adjusted to experimental requirements. For ICC and cell viability assays, 10,000 OLs or microglia and 250,000 astrocytes were seeded per well and in Ibidi μ‐Dishes for time‐lapse imaging. Myelin was added at 1 DIV.

### 2.4 Hippocampal Neuronal Cultures

Hippocampal neurons were prepared from embryonic day 18 rat embryos. Hippocampi were dissected from embryonic brains and dissociated in TrypLE Express (Thermo‐Fisher) for 10 min at 37°C. Cells were resuspended and homogenized in Neurobasal (Gibco) with 10% FBS Hyclone, 2 mM L ‐glutamine (both from Sigma‐Aldrich) and 50 U/mL penicillin–streptomycin (Gibco). Hippocampal neurons were cultured on PDL‐coated coverslips at 20.000 cells/well. The medium was supplemented with B27 (Gibco) and 20 μM 5‐fluorodeoxyuridine and uridine (Sigma Aldrich) and changed every 3 days. Myelin was added at 7 DIV.

### 2.5 Myelin Extraction and Labelling

Myelin was isolated from adult rat or mouse brains following the protocol established by Norton and Poduslo (1973). Briefly, brain tissue was mechanically homogenized in 0.32 M sucrose and subjected to rounds of ultracentrifugation using 0.85 M sucrose gradients. Concentration of isolated myelin was determined using Bradford protein assay (ThermoFisher) and labeled with Alexa488‐NHS or Alexa594‐NHS dye (Invitrogen) for 1 h at room temperature in PBS (pH = 8). Excess dye was removed by 24‐h dialysis, and labeled myelin was resuspended in sterile PBS (pH = 7.4) and frozen at −80°C.

For in vitro assays, myelin was thawed and vortexed for 30 s to obtain uniformly sized aggregates and added to culture medium at 5 μg/mL for immunofluorescence and cell viability experiments and 1 μg/mL for time‐lapse recordings. When required, cells were incubated with the following compounds: etomoxir (25 μM, E‐1905 Sigma Aldrich) and/or a mixture of fatty acids, referred to as “FA mix” composed of oleic acid (29557, Cayman Chemical), palmitic acid (29558, Cayman Chemical), linoleic acid (L9530, Sigma‐Aldrich), and arachidonic acid (34931, Cayman Chemical) at 1 μM each. For in vivo experiments, myelin was sonicated for 30 min before use and injected at 200 μg/mL.

### 2.6 Cell Viability Assays

Cell viability was assessed using the Calcein‐AM dye (Invitrogen). Cells were incubated with 1 μM of dye for 30 min and fluorescence was measured with a Synergy HT fluorimeter reader (Bio‐Tek Instruments). Results are expressed as the relative percentage of cellular viability with respect to control conditions.

### 2.7 Time‐Lapse Imaging

Alexa‐488 labeled myelin was added to the culture media 15 min prior to the beginning of the recording to let myelin debris settle and avoid out‐of‐focus images. Bright‐field and fluorescence images (488 nm laser) were acquired every 10 min in 8–10 localized points using a BioStation IM‐Q microscope (Nikon). Cells were maintained at 37°C and 5% CO_2_ during the recording.

### 2.8 Immunochemical Analysis

For ICC analysis, primary cultures were fixed in 4% paraformaldehyde (PFA) diluted in 0.1 M phosphate buffer (PB) for 20 min. IHC was performed in free‐floating sections obtained as described in section 12.2. A standard immunofluorescence protocol was used consisting of a 1 h incubation at room temperature (RT) in blocking solution containing 4% normal goat serum (Vector Labs) and 0.1% triton X‐100 in PBS, except for PDGFR‐α antibody for which normal donkey serum was used. Cells were incubated overnight at 4°C with primary antibodies, followed by an hour incubation at RT with AlexaFluor‐conjugated secondary antibodies and DAPI (4 μg/mL; Sigma‐Aldrich) for nuclei staining. After washings, coverslips were mounted on glass slides using Dako Glycergel Mounting Medium (Agilent).

Primary antibodies used for immunohistochemistry were as follows: mouse anti‐MBP (1:500; Biolegend), goat anti‐PDGFR‐α (1:250; R&D Systems), rabbit anti‐NG2 (1:500; Abcam), mouse anti‐Olig2 (1:500; Milipore), rabbit anti‐Ki67 SP6 (1:250; Abcam), and guinea pig anti‐Iba1 (1:300; Synaptic Systems). The corresponding Alexa Fluor secondary antibodies (ThermoFisher) were used according to the host species of primary antibodies at a 1:500 dilution.

### 2.9 Lipid Droplet Staining and Imaging

Fixed cells were stained with Oil Red O (BioVision), following manufacturer's instructions: cells were incubated in 60% isopropanol for 5 min and then stained with Oil Red O (1.8 mg/mL in dH_2_O) for 20 min. After consecutive washings in dH_2_O, nuclei were stained with DAPI (4 μg/mL; Sigma‐Aldrich) and coverslips were mounted. Images were acquired in a Zeiss LSM880 Airyscan microscope using a 40× objective.

### 2.10 Confocal Imaging and Analysis

Immunofluorescence imaging was conducted on a Zeiss LSM880 Airyscan or Leica TCS SP8. Imaging parameters were kept constant within each experimental set. Image processing and quantification were carried out using FIJI (ImageJ) software.

To assess oligodendrocyte morphology, we performed Sholl analysis. Individual cells were segmented as regions of interest and skeletonized. Morphological complexity was measured as the number of intersections between cell processes and concentric circle templates centered on the soma.

### 2.11 Bulk RNA Sequencing

Total RNA from OLs cultures was isolated using NZY Total RNA Isolation kit (NZYTech), according to the manufacturer's instructions. The quantity and quality of the RNAs were evaluated using Qubit RNA HS Assay Kit (Thermo Fisher) and Agilent RNA 6000 Nano Chips (Agilent Technologies), respectively. Sequencing libraries were prepared following “TruSeq Stranded mRNA Sample Preparation Guide (Part # 15031058 Rev. E)” using the “TruSeq Stranded mRNA Library Prep” kit and TruSeq RNA CD Index Plate (Illumina). From 400 ng of total RNA, mRNA was purified, fragmented and primed for cDNA synthesis using Illumina and ThermoFisher reagents. Then, A‐tailing and adaptor ligation were performed. Finally, enrichment of libraries was achieved by PCR and quantified using Qubit dsDNA HS DNA Kit (Thermo Fisher Scientific) and visualized on an Agilent 2100 Bioanalyzer using Agilent High Sensitivity DNA kit (Agilent Technologies). Using STAR software version 2.7.10b (Dobin et al. 2012), FASTQ files were aligned to *Rattus norvegicus* genome data base “rn6” and reads for analyzed features were assigned and counted from the processed BAM files using SubRead's FeatureCounts version v2.0.3 (Liao et al. 2014). Differential expression analysis was performed with the R library DESeq2 version 1.44.0 (Love et al. 2014). GSEA were performed with described gene sets using gene set permutations (*n* = 1000) for the assessment of significance and signal‐to‐noise metric for ranking genes from the Molecular Signature Database (https://www.gsea‐msigdb.org/gsea/msigdb/index.jsp) (Liberzon et al. 2015).

### 2.12 Exogenous Myelin Internalization in Mice

#### 2.12.1 Stereotaxic Injections

Brain stereotaxic injections were performed in 8–10 weeks old C57BL/6J mice. Two 0.5 μL injections of mouse‐derived Alexa488‐labeled myelin diluted in saline solution (0.9% NaCl) were performed at the following coordinates: Bregma 0.5 mm AP, 1.0 mm LM, 1.3 mm VD and Bregma 2.5 mm AP, 0.8 mm LM, 1.0 mm VD. The content was injected at a constant rate of 0.1 μL/min using a Hamilton syringe, leaving the needle in place for an additional 3 min. Both injections were made in the same hemisphere. In sham‐operated control mice, saline was injected using the equivalent procedures. Animals were perfused 24 or 48 h after the injection.

#### 2.12.2 Tissue Processing for Immunohistochemistry

Mice were anesthetized and perfused with 4% PFA in 0.1 M PB. Dissected brains were post‐fixed overnight at 4°C in the same solution. Then, coronal 40 μm‐thick sections were obtained using a Microm HM 650 V Microtome (ThermoFisher). Sections were selected for further staining based on the presence of fluorescent signal from myelin and/or the tissue scar from the surgery in the case of sham animals.

#### 2.12.3 Transmission Electron Microscopy

Mice were anesthetized and perfused with 4% paraformaldehyde and 0.1% glutaraldehyde in 0.1 M PB. Coronal 50 μm‐thick brain sections were obtained using a Microm HM650V microtome (Thermo Fisher). To stain Alexa‐488 labeled myelin using immunogold, slices were incubated with blocking solution (10% BSA, 0.1% sodium azide, 0.05% triton X‐100 in TBS) for 60 min at RT. Subsequently, sections were incubated with rabbit IgG anti‐Alexa 488 antibody (1:100, ThermoFisher) in blocking solution for 4 days at 4°C. Following several washes, sections were incubated for 2 h at RT with 1.4 nm gold‐labeled goat anti‐rabbit IgG (1:200; Nanoprobes Inc.) in blocking buffer. Sections were postfixed in 1% glutaraldehyde for 10 min at RT and washed in ddH20. Gold particles were silver‐intensified with a HQ Silver kit (Nanoprobes) in the dark for 12 min and tissue was washed with ddH20 followed by 0.1 M PB. The day after, sections were osmicated (1% OsO4 in 0.1 M PB) for 30 min and dehydrated in graded ethanol concentrations (50–100%) to propylene oxide and embedded in epoxy resin (Sigma‐Aldrich) by immersion in decreasing concentration of propylene oxide. Tissue was then embedded in fresh resin overnight and allowed to polymerize at 60°C for 2 days. Semithin sections (500 nm thick) were cut using a PowerTome ultramicrotome (RMC Boeckeler) and stained with 1% toluidine blue for sample orientation. Ultrathin sections (50–60 nm thick) were cut with a diamond knife (Diatome), collected on nickel mesh grids, and stained with 4% uranyl acetate for 30 min followed by 2.5% lead citrate for 10 min. TEM images were obtained using the JEOL JEM 1400 Plus electron microscope (SGiker, UPV/EHU) at magnifications ranging from 1000× to 8000×. Specificity of Alexa labeling in EM was corroborated by the absence of signal in control animals (Figure S6).

### 2.13 Myelin Internalization in Zebrafish

#### 2.13.1 Intracerebroventricular Injections

At 3 dpf, larvae were anesthetized using 600 μM Tricaine (Sigma‐Aldrich) and embedded in a 2.5% agarose drop. Larvae were oriented ventrally, with the yolk sac oriented to the bottom of the drop, allowing access to the dorsal area. The needle was positioned through the thin roof plate of the hindbrain without damaging the brain tissue beneath. Each larva received two sequential 0.5 nL microinjections of Alexa‐594 or −488 labeled myelin in nuclease‐free water, which was also injected as a control. Following the injection, heartbeat and circulation were checked and larvae were gently released from the agarose. Injected fish were transferred to 12‐well plates with E3, where they were maintained until imaging. Fish were monitored until full recovery from anesthesia, and only those exhibiting normal swimming behavior were included in subsequent analyses.

#### 2.13.2 Live Imaging and Quantification of Zebrafish Larvae

For live imaging, 3–4 dpf zebrafish larvae were anesthetized with 600 μM Tricaine (Sigma‐Aldrich) and mounted laterally in 2.5% agarose as described by Vagionitis and Czopka (2018). Imaging was performed using a Zeiss LSM880 confocal microscope equipped with Airyscan detection and a Zeiss W Plan‐Apochromat 20×/1.0 NA water‐dipping objective or Zeiss Axio Imager Z1 equipped with an Apotome.2 unit and using a 10× objective. Larvae injected with labeled myelin were visually scored for distribution of fluorescence over the spinal cord area as high (continuous fluorescence > 10 clusters), low (sparse < 5 clusters), or medium with intermediate amounts of fluorescence. Animals lacking detectable fluorescence were excluded from experimental groups.

For internalization experiments, fluorescence was assessed throughout the spinal cord and brain, with specific regions of interest sampled for analysis. For figure preparation, maximum‐intensity projections of z‐stacks were generated and representative regions were cropped.

For cell counting, the anal pore was used as a reference point to image a consistent anterior region covering the full depth of the spinal cord. Individual oligodendrocytes were manually counted in each z‐stack and classified as ventral or dorsal based on their contact with the Mauthner axon and normalized to the imaged length. A minimum of two independent injection rounds was analyzed. All zebrafish images are shown as lateral views of the spinal cord, with anterior to the left and dorsal at the top.

### 2.14 Statistical Analysis

Data are presented as mean ± standard error of mean (SEM) and n represents the number of animals, cultures or cells analyzed, as specified in figure legends. Statistical analyses were performed using GraphPad Prism 8 (GraphPad Software Inc). Comparisons between two groups were analyzed using paired Student's two‐tailed *t*‐test in in vitro experiments and unpaired in in vivo experiments. *p* values < 0.05 were considered statistically significant.

## 3 Results

### 3.1 Oligodendroglia Internalize Exogenous Myelin Debris In Vitro

**FIGURE 1:**
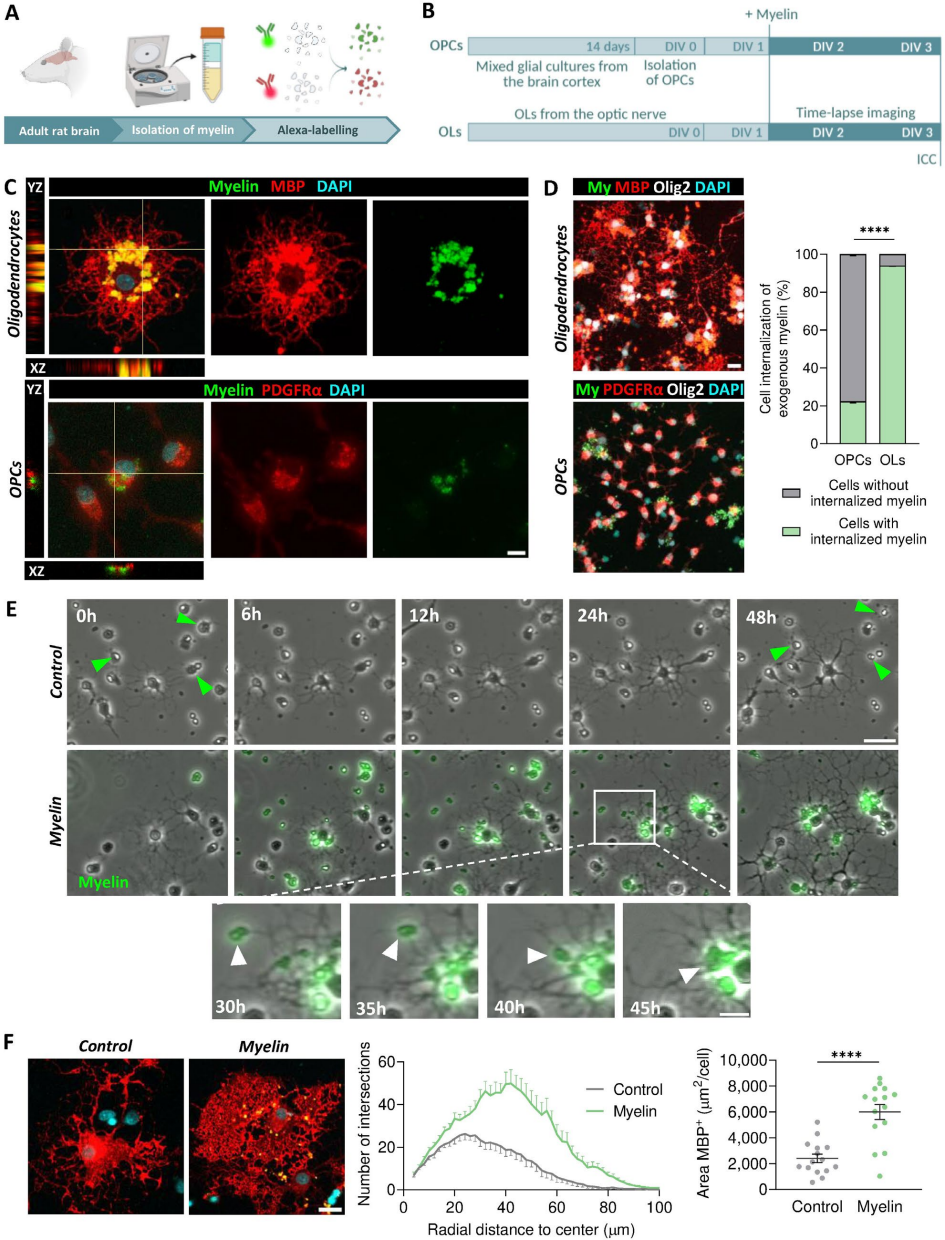
Internalization of exogenous myelin by oligodendroglial cells. (A) Schematic illustrating the isolation and fluorescent labelling of myelin debris. (B) Experimental design for evaluating myelin uptake. (C) Orthogonal confocal views showing internalized exogenous myelin within oligodendrocytes (OLs) and oligodendrocyte precursor cells (OPCs). Scale bar: 10 μm. (D) Comparative analysis of myelin internalization between OPC and OL cultures. Scale bar: 20 μm. (E) Time‐lapse imaging of OLs reveals increased morphological complexity and cell abundance in response to myelin exposure (top), and transport of internalized myelin along OL processes (bottom). Scale bar: 50 μm; zoomed‐in panel: 20 μm. (F) Sholl analysis of OLs after 48 h of myelin exposure. Scale bar: 20 μm. Area under the curve: Control, 1052 ± 78.7; Myelin, 2303 ± 139; ****p < 0.0001. Peak number of intersections: Control, 26.2; Myelin, 49.9. Each dot represents a single cell (*n* = 15).

In order to address the internalization capacity of oligodendroglial cells along the lineage, we first exposed primary rat cell cultures to Alexa‐labeled myelin debris for 48 h (Figure 1A,B). Myelin was isolated from rats (Figure 1A) to prevent cross‐species reactivity. Notably, both OLs and OPCs were found to internalize exogenous myelin (Figure 1C). Confocal imaging of cells labeled with the membrane‐linked Calcein‐AM dye confirmed the internalization of myelin debris by oligodendroglia (Figure S1).

Interestingly, our results suggest a maturation‐dependent enhancement of phagocytic or endocytic activity within the oligodendroglial lineage (Figure 1D). Specifically, OLs were more efficient at engulfing extracellular myelin, with more than 90% of cells containing internalized myelin particles. In contrast, OPCs presented significantly lower levels of internalization, with only about 20% of the cells showing uptake, potentially indicating a more limited role in debris clearance at this developmental stage.

In order to further explore the temporal dynamics of myelin internalization, we performed time lapse imaging of cultured OLs over 48 h (Figure 1E). Interestingly, the processes of OLs were the first cellular region contacting the myelin debris and some particles were observed migrating along the processes towards the soma (Figure 1E, bottom section). After 48 h, myelin accumulated predominantly in clusters within the cytoplasm and only small particles remained in the processes. In phagocytic cell types, such as microglia and macrophages, internalized material typically accumulates in the soma, where it is processed and degraded by lysosomal enzymes (Trivedi et al. 2020; Yu et al. 2022).

During the 48‐h time‐lapse imaging period, OLs remained static while their processes extended radially. Notably, OL processes did not exhibit directed growth towards the nearby myelin particles, suggesting that they do not actively pursue it. Instead, they appear to maintain their intrinsic growth patterns and may initiate internalization upon passive contact. Importantly, compared to controls, OLs exposed to myelin appeared more ramified and exhibited an increased cell size. Morphological Sholl analysis confirmed that myelin‐exposed OLs were larger and exhibited higher process complexity, as determined by increased branching (Figure 1F). Moreover, exposure to myelin enhanced OL survival, as evidenced by reduced cell death compared to control conditions (Figure S2).

Building on these observations, we next investigated the effects of myelin exposure on microglia, astrocytes, and neurons in vitro. As expected, microglial cells (Figure S3A) actively phagocytosed myelin particles and over 1 h most of the microglia cell bodies visible by phase contrast progressively became Alexa‐Fluor^+^. The microglial response was characterized by a higher and more rapid uptake of myelin when compared to OLs, supporting their established role in the clearance of myelin debris as professional phagocytic cells (Franklin and Ffrench‐Constant 2017; Kotter et al. 2006). In contrast, astrocytes (Figure S3B) exhibited minimal uptake, and some myelin clusters were observed around the cells. Moreover, exposure to myelin did not appear to disrupt the astrocytic monolayer. Similarly, neurons showed no significant internalization of myelin (Figure S3C). In both cases, exposure to myelin did not affect cellular survival (Figure S3D).

These findings highlight the distinct contributions of different cell types in the internalization and clearance of myelin debris. Moreover, myelin appears to signal back specifically to OLs, promoting lineage progression and survival. These results lead us to hypothesize that myelin debris may alter metabolic and signaling pathways, thereby promoting OL differentiation and/or myelination. Our results demonstrate that OLs, in addition to microglia, are also capable of internalizing myelin, particularly as they mature. Based on these findings, we directed subsequent experiments towards myelinating OLs to further elucidate pathways triggered by myelin internalization.

### 3.2 Exposure to Myelin Alters the Metabolic Transcriptional Profile of Oligodendrocytes

**FIGURE 2:**
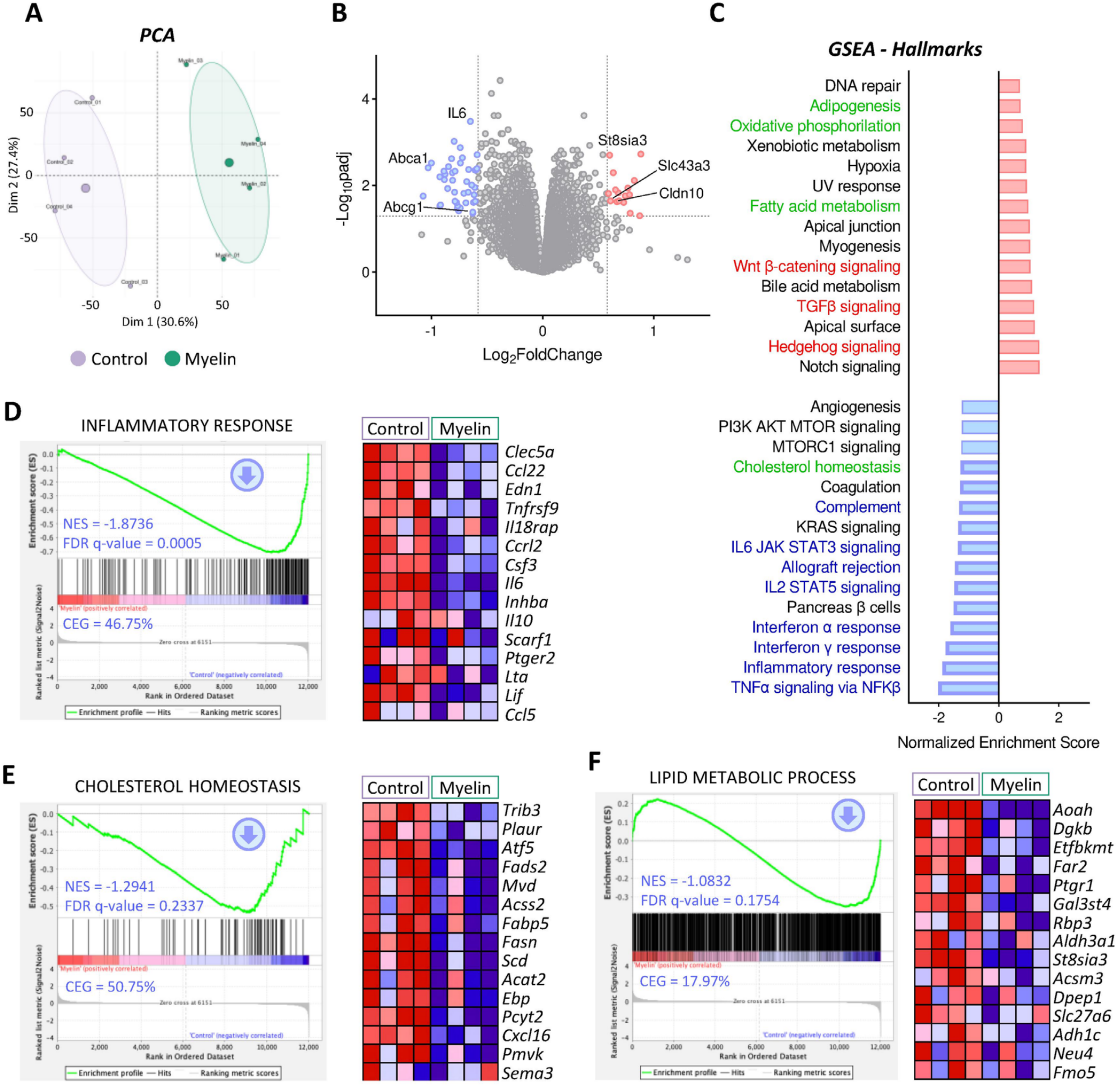
Transcriptional profile of oligodendrocytes exposed to exogenous myelin debris. OLs were treated with Alexa‐labeled myelin for 48 h, from DIV 1 to 3, followed by transcriptomic analysis (*n* = 4 cultures). (A) Principal component analysis (PCA) showing clear separation between treated and untreated samples. (B) Volcano plot displaying significantly upregulated genes (red) and downregulated genes (blue) in OLs exposed to myelin compared to control conditions. Differentially expressed genes (DEGs) were defined by |fold change| > 1.5 and adjusted *p* value < 0.05. Representative DEGs are labeled. (C) Gene Set Enrichment Analysis (GSEA) of Hallmark pathways, showing the top 15 upregulated (red) and downregulated (blue) biological processes ranked by normalized enrichment score (NES). Significantly enriched pathways (FDR *q* value < 0.25) are highlighted. Metabolic, differentiation‐related and immune‐related pathways are indicated in green, red, and blue, respectively. (D, E) Enrichment plots (left) for selected Hallmark gene sets with corresponding NES, FDR *q* values, and Core Enrichment Genes (CEGs), defined as the percentage of significantly enriched genes in the set. Heatmaps (right) show the top 15 most enriched genes for each pathway. (F) Enrichment plot for the “Lipid metabolic process” gene signature.

We performed RNA sequencing of OLs cultured in the presence of myelin for 48 h. Volcano plot depicted subtle changes, with 63 differentially expressed genes (Figure 2B). Gene Set Enrichment Analysis (GSEA) (Figure 2C) revealed that myelin exposure triggered a downregulation of immune‐related signaling, including tumor necrosis factor alpha (TNFα), interleukin signaling, complement pathway, as well as interferon and inflammatory response (Figure 2D). This transcriptomic profile suggests a distinct cellular phenotype, diverging from the disease‐specific state activated in OLs under experimental MS conditions (Falcão et al. 2018).

**FIGURE 3:**
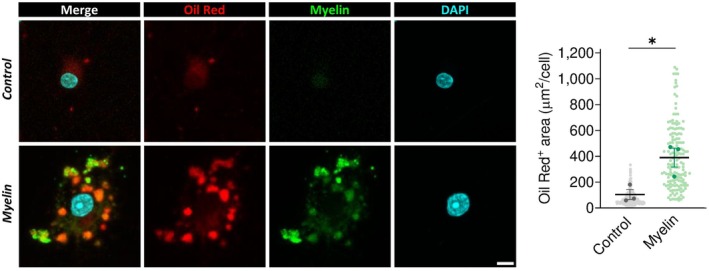
Lipid transport and storage following myelin internalization in oligodendrocytes. OLs were cultured with Alexa Fluor 488‐labeled myelin for 48 h. Myelin internalization led to increased formation of lipid droplets, visualized by Oil Red O staining, which labels neutral lipids. Scale bar: 10 μm. *p < 0.05.

In addition, several metabolic pathways were significantly altered. Notably, genes involved in cholesterol homeostasis were downregulated (Figure 2E). This included reduced expression of key enzymes critical to the mevalonate pathway for cholesterol biosynthesis, such as mevalonate diphosphate decarboxylase (*Mvd*), phosphomevalonate kinase (*Pmvk*), and emopamil binding protein (*Ebp*), along with acyl‐CoA synthetase short‐chain family member 2 (*Acss2*), which contributes to precursor synthesis. These findings suggest that internalized myelin may serve as an exogenous source of cholesterol, thereby reducing the need for *de novo* synthesis and potentially facilitating lipid recycling for new myelin production. Indeed, Oil Red O staining revealed that internalized myelin induced an increase in lipid droplets (LDs) formation in OLs, which colocalize with myelin, suggesting that internalized myelin is stored within lipid droplets (Figures 3 and S4). LDs are key organelles that maintain lipid homeostasis, prevent cholesterol overload (Nugent et al. 2020; Ralhan et al. 2021), and regulate lipid intermediates necessary for myelin synthesis (Berghoff, Spieth, Sun, Hosang, et al. 2021; Berghoff, Spieth, Sun, Ischebeck, et al. 2021; Hayashi and Su 2004). Therefore, the presence of LDs in our model further supports the notion that internalized myelin is actively being processed and recycled.

We also found a downregulation of genes involved in fatty acid metabolism within the “Cholesterol Homeostasis” gene signature. This included fatty acid synthase (*Fasn*), a key enzyme involved in *de novo* fatty acid synthesis, along with stearoyl‐CoA desaturase (*Scd*) and fatty acid desaturase 2 (*Fads2*), which mediate fatty acid desaturation and elongation, respectively. Additionally, genes linked to lipid anabolism within the “Lipid Metabolic Process” signature, such as fatty acyl‐CoA reductase 2 (*Far2*) and fatty acid transporter 6 (*Slc27a6*), were also downregulated (Figure 2F). Moreover, pathways associated with lipid catabolism showed a modest trend towards enrichment, including those related to fatty acid metabolism and oxidative phosphorylation (Figure S5A,B). These findings suggest that myelin‐derived metabolites may modulate the lipid pool in OLs, thereby downregulating endogenous lipid biosynthesis pathways.

Finally, pathways associated with cell proliferation and differentiation, such as wingless‐related integration site β‐catenin (*Wntβ*) (Figure S5C) and transforming growth factor β (*Tgfβ*) signaling (Figure S5D), exhibited a small, non‐significant enriched expression. The “Oligodendrocyte differentiation” signature (Figure S5E) showed a similar pattern. Paradoxically, late differentiation markers of OL (*Mbp, Plp1*, or *Mag*) were not differentially expressed, and the top upregulated genes within the “Oligodendrocyte differentiation” signature were primarily associated with OPC proliferation, including *Clip3, Lsm11, Rad54b*, and *Rcor2*. Altogether, the transcriptomic analysis revealed that myelin exposure upregulates specific metabolic and signaling pathways that may facilitate oligodendroglial lineage progression.

### 3.3 Myelin Internalization Triggers Oligodendrocyte Proliferation In Vitro

**FIGURE 4:**
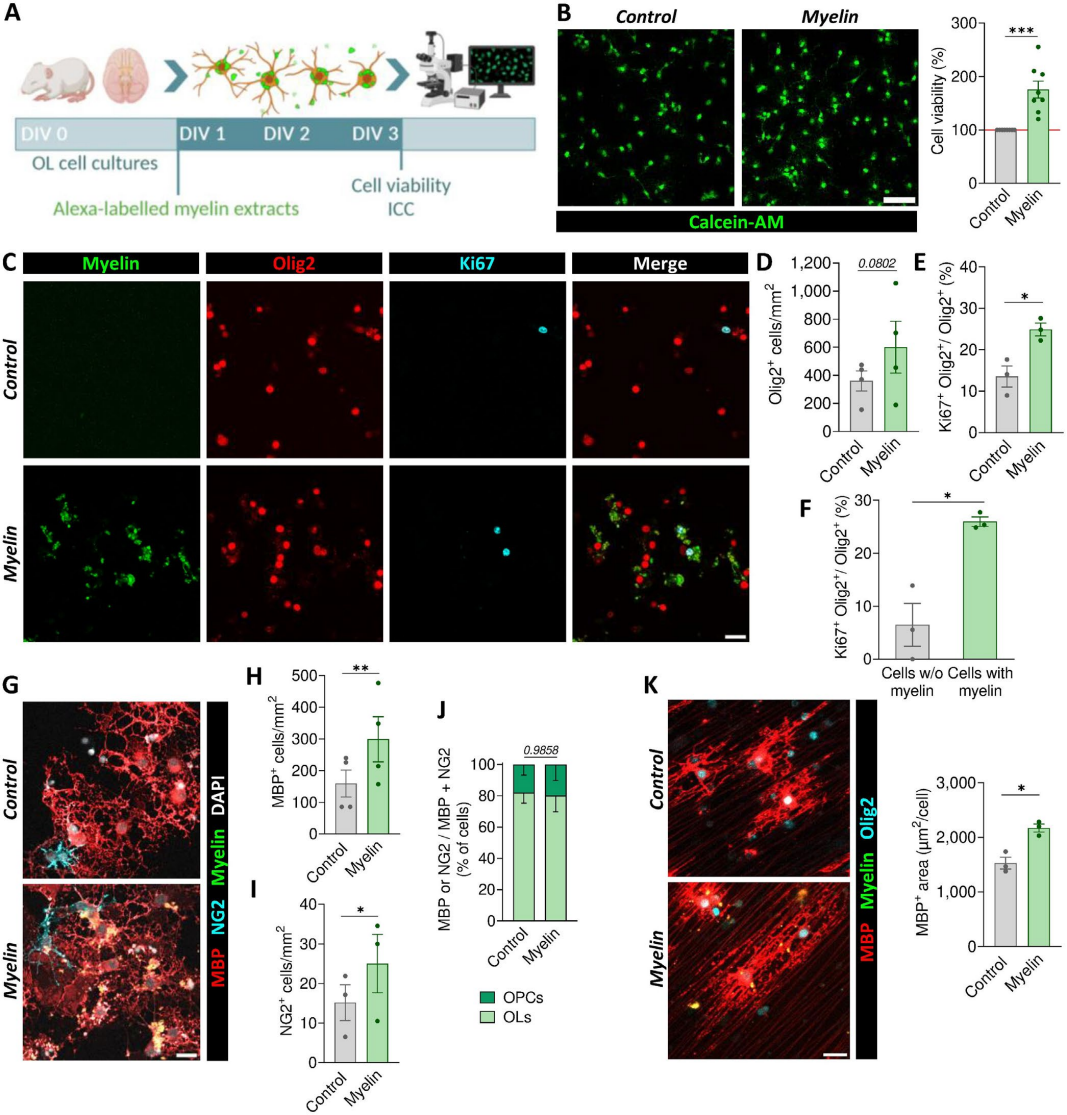
Exogenous myelin promotes oligodendroglial lineage progression. (A) Schematic representation of the experimental design. (B) Calcein‐AM viability assay showing fluorometric values normalized to control mean. (C) Immunocytochemical analysis of OLs cultures indicates that myelin exposure increases the total number of Olig2^+^ OLs (D) and the number of Ki67^+^ Olig2^+^ proliferating OLs (E). (F) Among myelin‐exposed OLs, proliferation rate, measured as the percentage of Ki67^+^ Olig2^+^, increases specifically in cells with internalized myelin debris. (G) Analysis of the different oligodendroglial populations also revealed that this increase results in higher numbers of (H) myelinating oligodendrocytes (MBP^+^) and (I) OPCs (NG2^+^), (J) without affecting the proportion of progenitors to mature OLs. (K) Enhanced myelination of nanofibers by myelin‐exposed OLs, reflected by an increased cellular area. Scale bars: (A–C) 50 μm; D‐E 25 μm. *p < 0.05, **p < 0.01, ***p < 0.001.

We next assessed the effects of myelin internalization on oligodendroglial lineage progression. After exposing primary OL cultures to myelin, we found that myelin debris increased cell viability by ~80%, as assessed by Calcein‐AM, which could be attributed either to a greater number of viable cells and/or to an increase in cell size (Figure 4B). To better understand the underlying cause, we conducted additional analyses using immunocytochemistry. Consistent with cell viability assays, immunostaining of the oligodendroglial lineage marker Olig2 confirmed an increase in the total number of cells (Figure 4C,D). Interestingly, proliferation marker Ki67 was enhanced in cultures exposed to myelin (Figure 4E), suggesting a boost in proliferation. Among myelin‐exposed OLs, proliferation was higher in cells that internalized myelin compared to those without internalized debris (Figure 4F). This observation likely reflects an expansion of the OPC pool (Dimou and Götz 2014; Young et al. 2013), which was further confirmed by the increase in NG2^+^ cells (Figure 4I). In parallel, we detected higher numbers of MBP^+^ myelinating oligodendrocytes (Figure 4G,H), while the ratio of NG2^+^ to MBP^+^ cells remained constant (Figure 4J). Thus, myelin internalization accelerates lineage progression maintaining the dynamics between precursor and mature stages. These changes prompted us to investigate whether internalized myelin boosted myelination capacity of mature OLs, typically associated with a more differentiated state (Bradl and Lassmann 2010). To this end, we seeded mature OLs onto coverslips containing synthetic nanofibers. We found that exposure to myelin significantly enhanced nanofiber myelination, as shown by an increase in OL area (Figure 4K).

**FIGURE 5:**
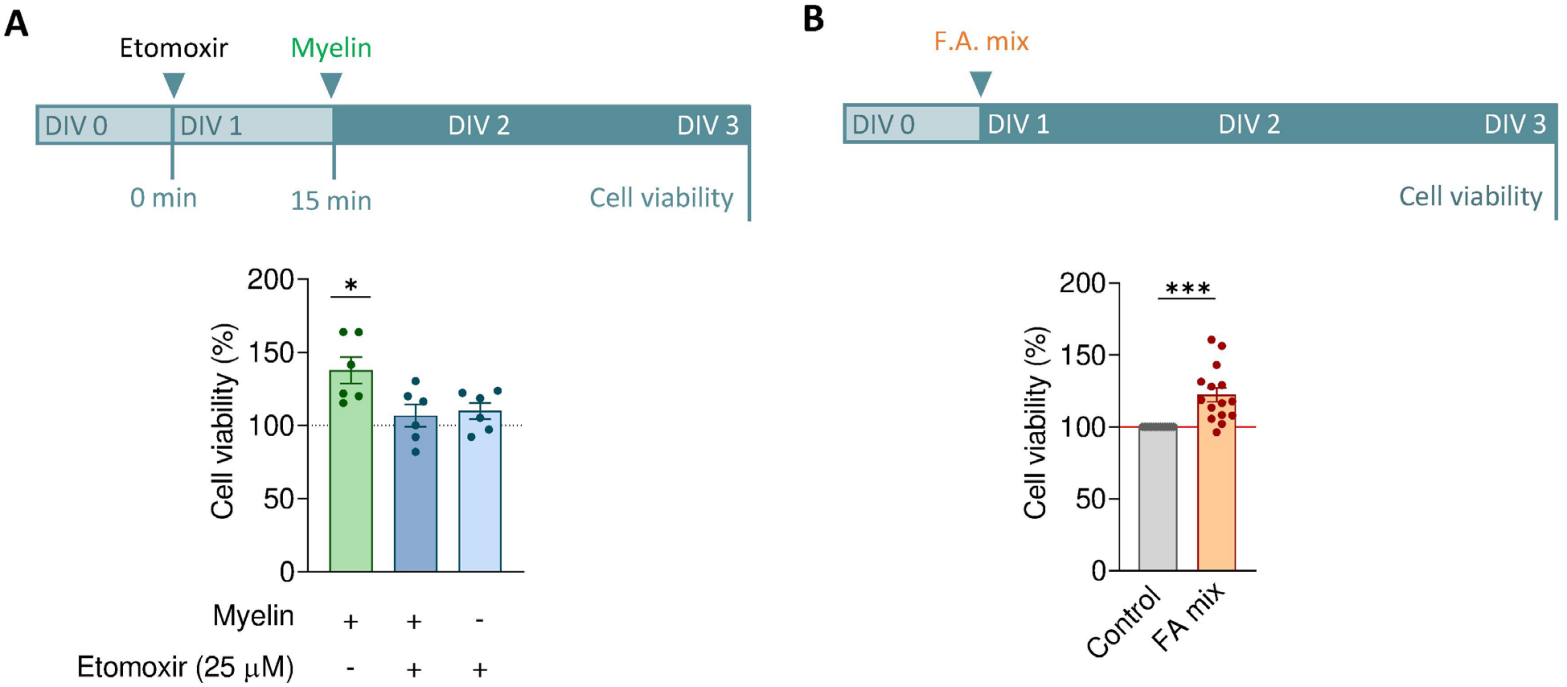
Contribution of myelin‐derived fatty acids to enhanced oligodendroglia viability. (A) Calcein‐AM assay demonstrating that inhibition of β‐oxidation with etomoxir blocks the myelin‐induced increase in cellular viability. (B) A mix of myelin‐derived fatty acids (arachidonic, linoleic, oleic and palmitic acid, 1 μM each; “FA mix”) partially mimics the viability enhancement observed with myelin. *p < 0.05, ***p < 0.001.

Lipids are the main constituent of myelin and have been previously suggested to act as an energy source and signaling molecules (Nave et al. 2023; Ramos‐Cabrer et al. 2025). To explore whether the effect of exogenous myelin is mediated by its lipid components, we examined the impact of etomoxir, an inhibitor of fatty acid oxidation. Pre‐incubation of OLs cell cultures with etomoxir abolished the myelin‐induced increase in cell viability, as measured using calcein assay (Figure 5A), suggesting a role for lipid metabolism in this process. Consistent with this, exposure to a mixture of four myelin‐derived fatty acids (arachidonic, linoleic, oleic, and palmitic acids, 1 μM each) also increased cell viability, although to a lesser extent than myelin (Figure 5B).

Taken together, our results suggest that myelin internalization triggers OL proliferation, as well as morphological complexity and maturation. Thus, myelin debris may serve as a positive regulator that activates the intrinsic program of OL development and myelination, promoting both expansion and progression of the oligodendroglial lineage.

### 3.4 Internalization of Exogenously Injected Myelin in the Mouse Brain

In vitro experiments were conducted in the absence of other cell types and their associated signaling interactions present in vivo. Therefore, it was important to investigate whether the internalization of myelin by OLs and the accompanying increase in proliferation was also observed in a more complex cellular environment. We aimed to determine first whether the capacity of OLs to internalize myelin is sufficiently robust to be observed in the presence of active microglia, and second whether microglial uptake of myelin debris triggers signaling cascades that modulate OL behavior. To this end, we performed stereotaxic injections of fluorescently labeled myelin into the cerebral cortex of adult mice. Although myelin debris is typically associated with demyelinating pathologies or injury, we sought to isolate the internalization process from confounding factors, such as OL dysfunction. While the stereotaxic procedure inherently induces a local inflammatory response, this approach enabled us to evaluate OL internalization capacity under non‐demyelinating conditions. Moreover, myelin turnover may also involve clearance mechanisms beyond microglial phagocytosis, potentially implicating OLs themselves.

**FIGURE 6:**
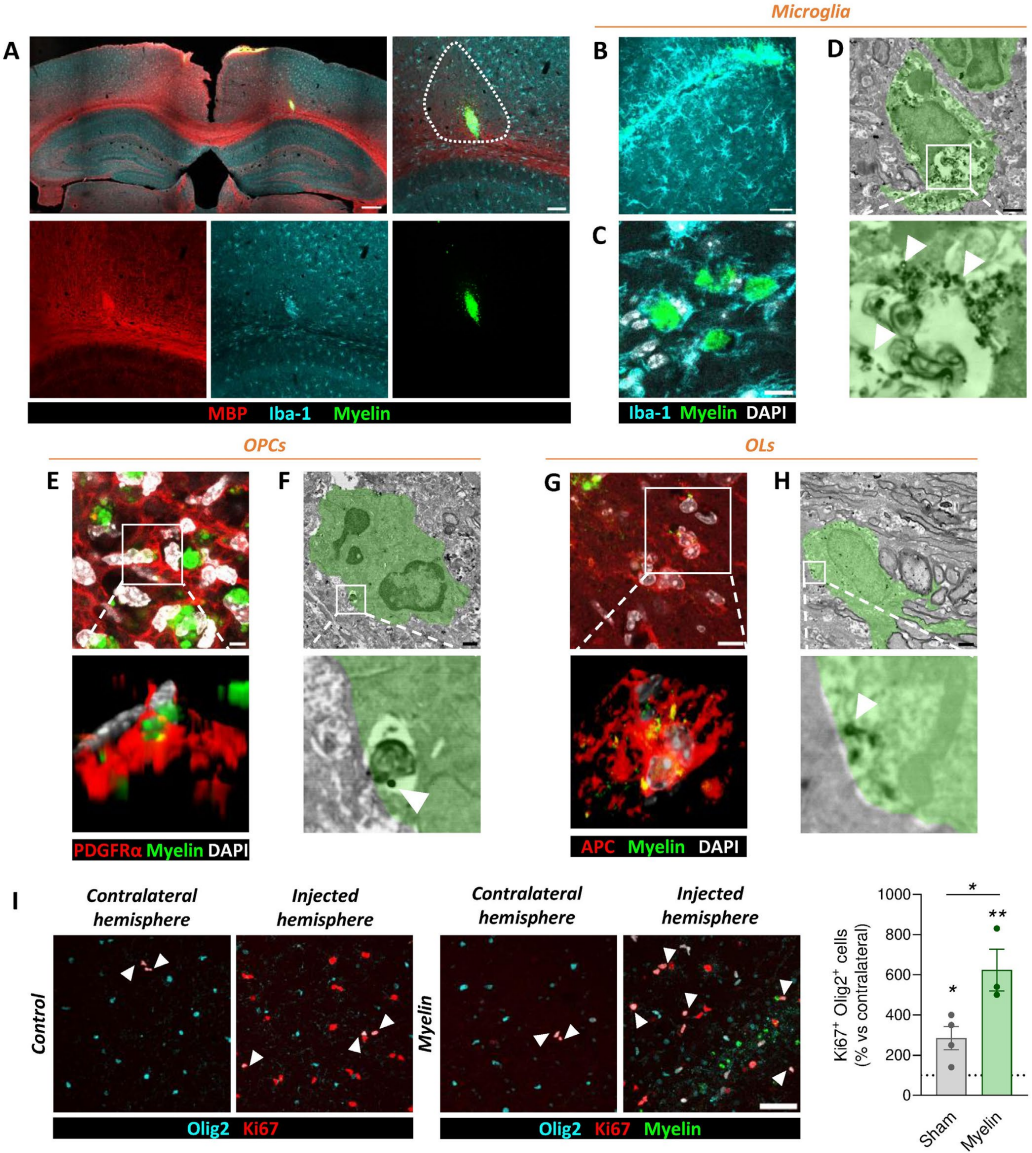
Internalization of Alexa Fluor 488‐labeled myelin debris in adult mice following stereotaxic injection. (A) Cortical section showing microglial migration to the injection site 24 h post‐surgery, resulting in a cell‐free gap around the injection core indicated by a dotted line. Scale bars: 300 μm (main), 100 μm (zoom). (B) Repopulation of surrounding areas by microglia 48 h after injection. Scale bar: 50 μm. (C) Formation of phagocytic pouches within microglia actively internalizing myelin debris. Scale bar: 10 μm. (D, F, H) Immunogold labelling of Alexa Fluor 488‐labeled myelin (white arrows) confirms internalization by (D) microglia, (F) OPCs and (H) OLs, as observed by transmission electron microscopy (TEM). Scale bar: 1 μm. (E, G) Internalization of exogenous myelin by OPCs (E) and OLs (G) in vivo. *Bottom*: 3D reconstructions of the cells shown in the insets. Scale bar: 20 μm (*top*). (I) Quantification of Ki67^+^ Olig2^+^ oligodendroglia 48 h after injection in both vehicle‐ and myelin‐injected animals. Quantification was performed in a radius of 300 μm around the lesion site and normalized for its contralateral hemisphere. A dashed line indicates mean basal proliferation levels in a corresponding area of the contralateral hemispheres. Each dot represents an individual injection. Scale bar: 50 μm. *p < 0.05.

As expected, microglial cells rapidly migrated towards the injection site. Within the first 24 h post‐injection, most microglial cells accumulated in the injection core (Figure 6A), temporarily depleting microglia from the immediate surroundings and forming a circular gap with a radius of 150–300 μm, which was subsequently repopulated over the following 24 h (Figure 6B). Immunohistochemistry confirmed that microglia were the predominant cell type within the injection site and internalized debris was clearly localized within phagocytic pouches (Figures 6C and S6B). Microglia adopted a rounded, amoeboid morphology characteristic of activated, phagocytic cells. These findings were corroborated by electron microscopy (EM), which visualized Alexa‐labeled myelin debris within intracellular compartments in microglial cytoplasm (Figure 6D). This expected behavior validated the approach and served as a positive control that the surgery procedure did not impair the capacity of phagocytic cells to internalize exogenous myelin.

Interestingly, 48 h post‐injection we also identified a small subset of PDGRα^+^ OPCs (Figures 6E and S6B) and APC^+^ OLs (Figures 6G and S6B) containing myelin particles, as confirmed by TEM analysis (Figure 6F,H). Unlike the well‐formed phagocytic vesicles seen in microglia, internalized myelin within OLs appeared in smaller, scattered inclusions without the formation of large degradation compartments. This could also reflect the lower myelin load within OLs, which may allow them to degrade and process debris without activating the same specialized degradation pathways observed in professional phagocytes.

We next examined OL proliferation and found that myelin injection increased proliferation of OLs (Figure 6I). Notably, the stereotaxic procedure itself induced a local proliferative response in control animals. Therefore, in order to address whether myelin internalization influences OLs proliferation in vivo without injury‐related confounds, we complemented our analysis using the zebrafish model.

### 3.5 Exogenously Injected Myelin Increases Oligodendrocyte Number in the Zebrafish

Zebrafish larvae offer a transparent and genetically tractable system allowing for high spatial–temporal resolution for live imaging (Chia et al. 2022; Doszyn et al. 2024). This model enables in vivo visualization of myelin internalization dynamics while preserving the complexity of a living vertebrate organism.

**FIGURE 7:**
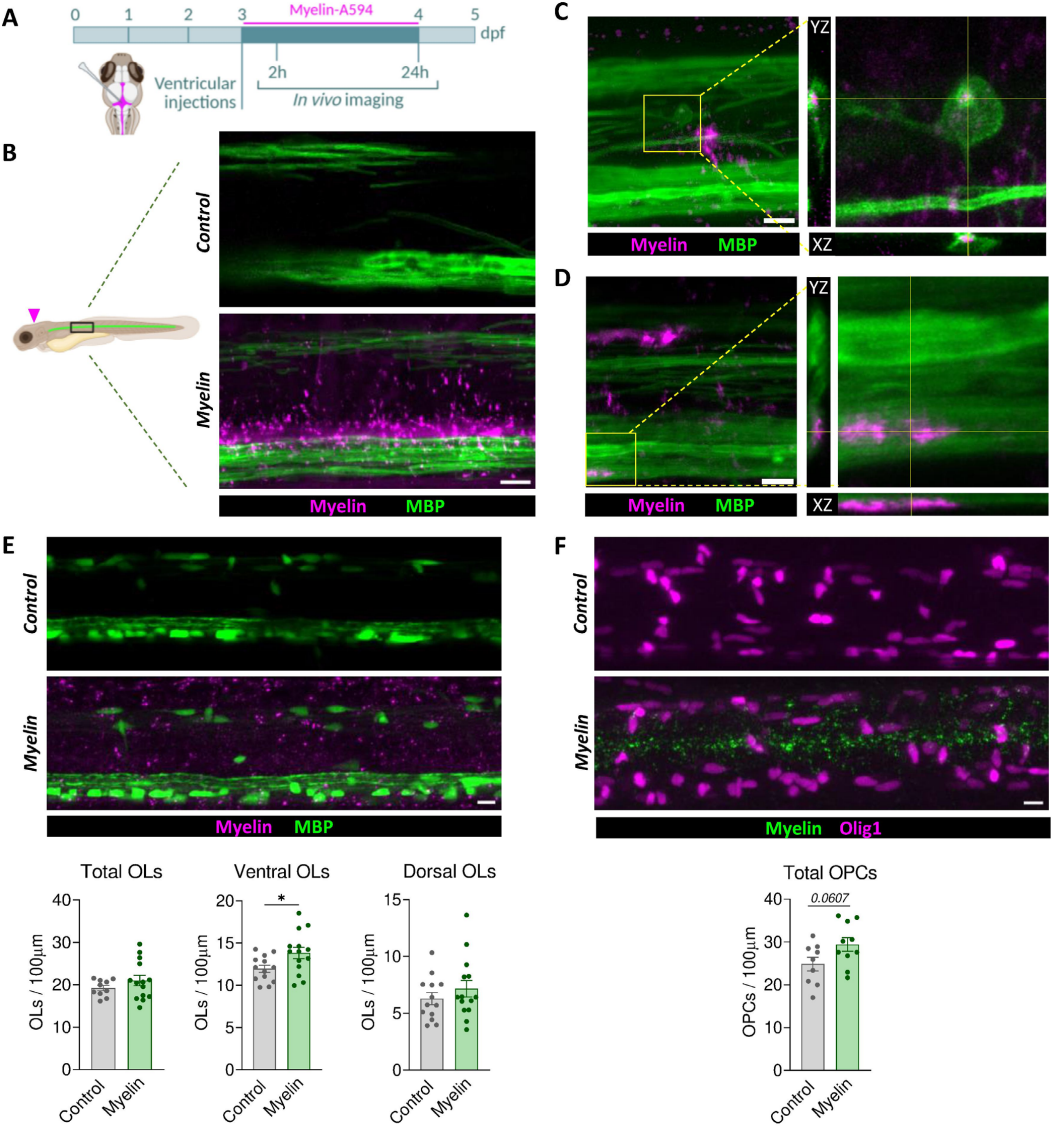
Oligodendrocytes internalize exogenous myelin in zebrafish. (A) Schematic representation of cerebroventricular injections of Alexa Fluor‐labeled myelin performed at 3 days post‐fertilization (dpf) in *Tg*(*mbp:EGFP‐CAAX*) zebrafish, which express membrane‐bound GFP in OLs. (B) Confocal imaging of the spinal cord 2 h post‐injection shows the diffusion of fluorescent myelin into the central canal in the anterior region. A pink arrow indicates the injection site. (C, D) 24 h post‐injection, myelin signal is largely cleared from the canal, but a small fraction of exogenous myelin remains visible within the soma (C) and processes (D) of OLs. (E) Representative spinal cord images from *Tg*(*mbp:EGFP*) (somatic fluorescence in OLs) and *Tg*(*Olig1:nls‐mApple*) (nuclear marker for OPCs) zebrafish at 24 h post‐injection, with quantification of myelin‐positive cells. Scale bar: 10 μm.

We performed brain ventricle injections in transgenic zebrafish larvae expressing membrane‐targeted EGFP under the myelin basic protein promoter (*Tg*(*mbp:EGFP‐CAAX*)), which allows selective labelling of OLs (Almeida et al. 2011). Membrane localization of EGFP facilitates the assessment of potential internalization of myelin, as engulfed debris can be clearly distinguished within the boundaries of myelinating oligodendrocytes, including fine processes and soma. Labeled myelin was injected into the brain ventricle at 3 days post‐fertilization (dpf) and, to avoid a potential confounding localized response to the injection, the spinal cord was imaged rather than the injection site at different time points (Figure 7A). At this developmental stage, brain ventricles are connected to the central canal, allowing the movement of injected material directly into the spinal cord. Control animals were injected with vehicle solution.

To determine the distribution and diffusion of Alexa‐labeled myelin following injection, we first imaged 2 h post‐injection. The spinal cord was divided into anterior, central, and posterior regions for analysis. The anterior region (Figure 7B), located closer to the brain ventricle, showed clear presence of labeled myelin debris dispersed along and outside the central canal, reaching both the ventral and dorsal myelinated tracts of the spinal cord. The central and posterior regions (Figure S7A), situated more caudally along the tail, showed little to no detectable myelin at this time point. This confirmed that the injected material diffused through the cerebrospinal fluid and reached the anterior spinal cord, allowing for potential interaction with resident oligodendrocytes. At this early time point, however, we did not observe internalization of myelin by OLs. Importantly, zebrafish larvae did not exhibit any visible behavioral abnormalities or morphological defects following injection and the introduced myelin was well tolerated, indicating that it was not toxic at the administered concentration.

By 24 h post‐injection, myelin fragments had formed more discrete clusters, typically 7–10 per fish, distributed along the spinal cord. To determine the identity of the cells containing these clusters, we repeated the injections in *Tg*(*mpeg1:EGFP*) zebrafish (Ellett et al. 2011), labelling microglia and macrophages. Colocalization analysis revealed that large myelin clusters were predominantly found within microglial cells, confirming their active role in myelin phagocytosis (Figure S7B). In addition to microglial uptake, we also detected smaller myelin particles contained within the soma of OLs (see example in Figure 7C) and associated with their myelin tracts (Figure 7D). These findings demonstrate that, consistent with our in vitro data and murine model, OLs in zebrafish are capable of internalizing exogenous myelin.

We next evaluated the impact of myelin internalization on the oligodendroglial lineage in the zebrafish spinal cord. Remarkably, 24 h after the injection of myelin, we observed a modest yet significant increase in the number of ventral OLs (Figure 7E), which are located closer to the central canal where the injected myelin accumulates. In contrast, dorsal OLs, potentially less exposed to myelin, did not show a significant change in cell number. This spatially restricted response suggests a direct, contact‐dependent effect, potentially driven by direct internalization of myelin debris. Moreover, OPCs, imaged in *Tg*(*olig1:nlsmApple*) zebrafish (Marisca et al. 2020), showed a non‐significant increase in number following injection of myelin (Figure 7F). Taken together, and considering that zebrafish development is characterized by low levels of oligodendroglial cell death (Almeida and Lyons 2016), these findings suggest that myelin uptake could locally be enhancing proliferation, rather than promoting oligodendroglial survival.

## 4 Discussion

In this study, we found that OLs have the capacity to internalize myelin debris, leading to transcriptional changes and promoting cellular proliferation and differentiation. Traditionally, the role of myelin debris in regulating oligodendroglial dynamics has been investigated in the context of demyelination, where it is known to inhibit OPC proliferation and/or differentiation (Franklin and Simons 2022; Kotter et al. 2006). However, the molecular mechanisms underlying this inhibitory effect remain incompletely understood (Baer et al. 2009). Moreover, demyelination involves massive tissue damage, microglia activation and immune infiltration. Thus, signaling from damaged tissue and glial cells could potentially affect the OL population (Ji et al. 2024; Molina‐Gonzalez et al. 2023). The deleterious impact of myelin debris is highly context‐dependent, shaped by the health of OLs and signals from the surrounding cellular environment. Our findings support the idea that, in the absence of overt pathology, myelin may enhance OL lineage progression.

Despite some insights, the extent to which OLs can internalize myelin and the underlying mechanisms remain insufficiently understood. Evidence suggests that OPCs may internalize myelin particles via phagocytosis (Falcão et al. 2018). Similarly, Schwann cells, the myelinating cells of the peripheral nervous system, have been shown to internalize myelin following nerve injury through specific autophagy pathways (Gomez‐Sanchez et al. 2015). In the case of myelinating OLs, several studies have demonstrated that, under pathological and physiological conditions, they express molecular components known to be involved in endocytic and phagocytic pathways. These include receptors such as low‐density lipoprotein receptor‐related protein 1 (LRP1), implicated in myelin uptake (Fernández‐Castañeda et al. 2020; Gaultier et al. 2009; Lin et al. 2017); antigen presentation molecules (Falcão et al. 2018; Zeis et al. 2015); and members of the tetraspanin family (Terada et al. 2002).

Our data show that exposure to myelin debris, in the absence of an inflammatory insult, induces an increase in OL proliferation and differentiation. These data could appear contradictory with the general concept that myelin debris injection (Kotter et al. 2006) or microglial deficiencies in myelin clearance during demyelination inhibit OPC recruitment, proliferation or differentiation, leading to impaired remyelination (reviewed in Franklin and Simons 2022). Nevertheless, the experimental paradigms in those previous studies also involved accumulation of myelin debris along with microglial activation, inflammation, and targeted OL damage (Blakemore and Franklin 2008; Lassmann and Bradl 2017; Torre‐Fuentes et al. 2020; Zirngibl et al. 2021), which inherently disrupts their function and may obscure broader roles. In response to injury, OPCs become activated, undergoing transcriptomic reprogramming to an immature state that promotes proliferation and migration by upregulation of immune‐related genes (Moyon et al. 2015). OLs also upregulate inflammatory mediators, stress response genes, anti‐oxidant enzymes, cholesterol metabolism and growth factors (Hou et al. 2023). This signature largely overlaps with the disease‐associated OLs recently reported in different neurodegenerative models (Pandey et al. 2022). Importantly, TREM2 deficiency in microglia impaired transcriptomic changes in disease‐associated OLs, suggesting that OL reprogramming is driven by microglial activation rather than by exposure to myelin debris (Hou et al. 2023). In contrast, we observed an opposite transcriptional signature in OLs after myelin uptake, characterized by a reduced expression of immune‐related pathways and cholesterol biosynthesis. These findings suggest that, in the absence of inflammation, myelin debris alone does not trigger a disease‐associated response in OLs, but rather promotes a distinct, non‐inflammatory program that may favor lineage progression and myelination.

In addition to experimental models of demyelination, some studies have investigated the impact of myelin on oligodendroglial cells in the absence of pathological cues (Baer et al. 2009; Plemel et al. 2013). However, the most common approach involved plating OPCs on coverslips with adhered myelin spots (Baer et al. 2009; Plemel et al. 2013), which restricts myelin availability and phagocytosis by OLs. Another important difference is that prior studies often employed myelin protein extracts rather than whole myelin debris (Baer et al. 2009; Plemel et al. 2013). This distinction may be critical, as we found that myelin‐derived enhancement of cell viability can be partially recapitulated by a combination of fatty acids. Therefore, differences in experimental design and myelin preparations could account for the apparent discrepancies among studies.

During CNS development, reduced expression of the cholesterol efflux via *Abca1* and *Abcg1* is associated with the onset of active myelination (Nelissen et al. 2011). Transcriptomic analysis also suggested predominant upregulation of the cholesterol‐synthesis pathway in OLs during remyelination (Voskuhl et al. 2019). This downregulation facilitates cholesterol retention within the cell, essential for myelin synthesis. A similar regulatory pattern emerges under demyelinating conditions, wherein neurons upregulate cholesterol synthesis, while OLs downregulate *Abca1* and *Abcg1* expression to minimize cholesterol efflux and prioritize lipid availability for remyelination (Berghoff, Spieth, Sun, Hosang, et al. 2021; Berghoff, Spieth, Sun, Ischebeck, et al. 2021; Meffre et al. 2015). Consistent with these observations, our findings demonstrate that when OLs are exposed to myelin debris, they adopt a lipid‐conserving phenotype that promotes cholesterol retention and supports myelin synthesis. Similarly, lipid droplets that regulate lipid intermediates necessary for myelin synthesis during development (Hayashi and Su 2007) are increased after myelin exposure. This adaptive response mirrors the molecular mechanisms observed during developmental myelination, suggesting a conserved strategy for optimizing lipid utilization.

While this study did not aim to dissect the molecular mechanisms through which myelin promotes OL proliferation and lineage progression, this remains a key area for future research. Based on its lipid‐rich composition and previous findings (Asadollahi et al. 2024; Ramos‐Cabrer et al. 2025), OLs could be using myelin to buffer energetic stress. Although the β‐oxidation capacity of OLs is considered limited (Fünfschilling et al. 2012; Rao et al. 2017; Tepavčevic 2021), there is evidence that suggests it is still functionally active (Hofmann et al. 2017). Importantly, in contrast to previous studies, our experimental paradigm did not restrict glucose availability. Therefore, the observed effects are unlikely to be driven by a metabolic shift away from their preferentially glycolytic activity, but rather as a response to excess lipid availability. Additionally, both lipids and proteins in myelin are known to exert signaling functions. Lipid receptors such as CPT1A (Tang et al. 2022), sphingosine‐1‐phosphate receptor (S1PR) (Jaillard et al. 2005) or lysophosphatidic acid receptor 1 (LPAR1) (Nogaroli et al. 2009; Yung et al. 2015) play essential roles in regulating lipid homeostasis and myelination. These pathways may be activated upon myelin internalization and could contribute to the observed proliferative and morphological responses in oligodendroglia.

Together with our zebrafish findings, these results suggest a fundamental principle of vertebrate nervous systems: that differentiated MBP^+^ OLs can internalize exogenous myelin under physiological conditions and respond to myelin debris by promoting the progression of the oligodendroglial lineage, opening new avenues for promoting remyelination and preserving CNS integrity in disease. The dynamics of this process and its extent across the OL population remain to be defined with further time‐course and time‐lapse studies.

## Author Contributions

Carla Peiró‐Moreno performed the majority of the experiments, analyzed and interpreted the data, and wrote the first draft of the manuscript. Juan Carlos Chara contributed to experimental design, performed key experiments in mice in vivo and electron microscopy, and participated in data analysis and interpretation. Katy Marshall‐Phelps performed specialized experiments in zebrafish and contributed to data acquisition and analysis. Irune Ugarte‐Arakistain and Stefano Calovi contributed to experimental work and data analysis. Rafael Gois De Almeida, María Domercq and Carlos Matute jointly conceived and designed and supervised the study, performed experiments, secured funding, contributed to data interpretation, and critically revised the manuscript.

## Funding

This work was supported by the Spanish Ministry of Science and Innovation (PID2019‐109724RB‐I00, PID2022‐143020OB‐I00, PID2022‐138276OB‐I00); Eusko Jaurlaritza (IT1551‐22); University of Edinburgh Chancellor's Fellowship to R.G.D.A. the Biotechnology and Biological Sciences Research Council (BB/X009394/1) and the UK Research and Innovation under the UK government's Horizon Europe funding guarantee (EP/Y024311/1).

## Ethics Statement

Mice work was approved by the internal Animal Ethics Committee of the University of the Basque Country (UPV/EHU). All zebrafish work was performed under UK Home Office regulations (PP0103366).

## Conflicts of Interest

The authors declare no conflicts of interest.

## Supporting information


**Figure S1:** Internalization of exogenous myelin by oligodendroglial cells. (A, B) Orthogonal confocal views showing exogenous myelin internalized within oligodendrocytes (A) and OPCs (B), visualized using Calcein‐AM to label live cells. Scale bar: 20 μm.
**Figure S2:** Oligodendroglia cell death in response to myelin. Representative images and quantification of cells labeled with propidium iodide (PI), which labels death cells, showed a higher cellular survival in OLs exposed to myelin debris. Scale bar: 20 μm. *p < 0.05.
**Figure S3:** Internalization of exogenous myelin by glial cells and neurons. (A–C) Time‐lapse imaging showing the internalization of myelin debris by microglia (A), astrocytes (B), and neurons (C). (D) Cell viability assay performed on each cell type following 48 h of exposure to myelin debris demonstrates no toxicity in all cell types. Scale bar: 10 μm.
**Figure S4:** Internalization of exogenous myelin by oligodendrocytes. OLs were cultured with Alexa Fluor 488‐labeled myelin for 48 h. The images show the double staining of lipid droplets (LDs), visualized by Oil Red O staining, with the oligodendroglial marker Olig2 to confirm the oligodendroglial identity of cells with LDs. Scale bar: 10 μm.
**Figure S5:** Metabolic and differentiation transcriptional profile of oligodendrocytes after myelin exposure. Enrichment plots (left) for selected Hallmark gene sets (A–D) or specific signatures (E) with corresponding NES, FDR q‐values and Core Enrichment Genes (CEG). Heatmaps (right) show the top 15 most enriched genes for each pathway.
**Figure S6:** Internalization of exogenous myelin in mice. (A) EM imaging of vehicle‐injected animals after immunogold labelling against Alexa‐488 did not show any unspecific labelling. (B) Quantification of total cells and cells within OPCs, OLs or microglia with internalized myelin 48 h post‐injection. Scale bar: 40 μm.
**Figure S7:** Internalization of exogenous myelin in the zebrafish model. (A) Confocal imaging of the spinal cord 2 h after cerebroventricular injection reveals the absence of fluorescent myelin in the central and posterior regions of the animal. (B) Microglia internalizing myelin debris 24 h post‐injection in the spinal cord of Tg(mpeg1:EGFP) zebrafish, which express EGFP in microglial cells. Scale bar: 10 μm.

## Data Availability

The data that support the findings of this study are available from the corresponding authors upon reasonable request.
