# Supplementary material for: Internalization of Exogenous Myelin by Oligodendroglia Promotes Lineage Progression
Source: Glia. 2026 Jan 7;74(3):e70132. doi: 10.1002/glia.70132 (PMC12775898; doi:10.1002/glia.70132)
Supplement: Supplementary file 1 — Figure S1: Internalization of exogenous myelin by oligodendroglial cells. (A, B) Orthogonal confocal views showing exogenous myelin internalized within oligodendrocytes (A) and OPCs (B), visualized using Calcein‐AM to label live cells. Scale bar: 20 μm. Figure S2: Oligodendroglia cell death in response to myelin. Representative images and quantification of cells labeled with propidium iodide (PI), which labels death cells, showed a higher cellular survival in OLs exposed to myelin debris. Scale bar: 20 μm. *p < 0.05. Figure S3: Internalization of exogenous myelin by glial cells and neurons. (A–C) Time‐lapse imaging showing the internalization of myelin debris by microglia (A), astrocytes (B), and neurons (C). (D) Cell viability assay performed on each cell type following 48 h of exposure to myelin debris demonstrates no toxicity in all cell types. Scale bar: 10 μm. Figure S4: Internalization of exogenous myelin by oligodendrocytes. OLs were cultured with Alexa Fluor 488‐labeled myelin for 48 h. The images show the double staining of lipid droplets (LDs), visualized by Oil Red O staining, with the oligodendroglial marker Olig2 to confirm the oligodendroglial identity of cells with LDs. Scale bar: 10 μm. Figure S5: Metabolic and differentiation transcriptional profile of oligodendrocytes after myelin exposure. Enrichment plots (left) for selected Hallmark gene sets (A–D) or specific signatures (E) with corresponding NES, FDR q‐values and Core Enrichment Genes (CEG). Heatmaps (right) show the top 15 most enriched genes for each pathway. Figure S6: Internalization of exogenous myelin in mice. (A) EM imaging of vehicle‐injected animals after immunogold labelling against Alexa‐488 did not show any unspecific labelling. (B) Quantification of total cells and cells within OPCs, OLs or microglia with internalized myelin 48 h post‐injection. Scale bar: 40 μm. Figure S7: Internalization of exogenous myelin in the zebrafish model. (A) Confocal imaging of the spinal cord 2 h afte [file GLIA-74-0-s001.docx]

******

***Supplementary Figure 1. Internalization of exogenous myelin by oligodendroglial cells.*** (**A-B**) Orthogonal confocal views showing exogenous myelin internalized within oligodendrocytes (**A**) and OPCs (**B**), visualized using Calcein-AM to label live cells. Scale bar: 20 µm.

***Supplementary Figure 2. Oligodendroglia cell death in response to myelin*.** Representative images and quantification of cells labelled with propidium iodide (PI), which labels death cells, showed a higher cellular survival in OLs exposed to myelin debris. Scale bar: 20 µm. *p < 0.05.

***Supplementary Figure 3. Internalization of exogenous myelin by glial cells and neurons***. (**A-C**) Time-lapse imaging showing the internalization of myelin debris by microglia (A), astrocytes (B), and neurons (**C**). (**D**) Cell viability assay performed on each cell type following 48 hours of exposure to myelin debris demonstrates no toxicity in all cell types. Scale bar: 10 µm.

***Supplementary Figure 4. Internalization of exogenous myelin by oligodendrocytes***. OLs were cultured with Alexa Fluor 488-labeled myelin for 48 hours. The images show the double staining of lipid droplets (LDs), visualized by Oil Red O staining, with the oligodendroglial marker Olig2 to confirm the oligodendroglial identity of cells with LDs. Scale bar: 10 µm.

***Supplementary Figure 5. Metabolic and differentiation transcriptional profile of oligodendrocytes after myelin exposure.*** Enrichment plots (left) for selected Hallmark gene sets (**A-D**) or specific signatures (**E**) with corresponding NES, FDR q-values and Core Enrichment Genes (CEG). Heatmaps (right) show the top 15 most enriched genes for each pathway.

***Supplementary Figure 6. Internalization of exogenous myelin in mice****. (***A**) EM imaging of vehicle-injected animals after immunogold labelling against Alexa-488 did not show any unspecific labelling. (**B**) Quantification of total cells and cells within OPCs, OLs or microglia with internalized myelin 48 hours post-injection. Scale bar: 40 µm.

***Supplementary Figure 7. Internalization of exogenous myelin in the zebrafish model****. (***A**) Confocal imaging of the spinal cord 2 hours after cerebroventricular injection reveals the absence of fluorescent myelin in the central and posterior regions of the animal. (**B**) Microglia internalizing myelin debris 24 hours post-injection in the spinal cord of *Tg(mpeg1:EGFP)* zebrafish, which express EGFP in microglial cells. Scale bar: 10 µm.
